# HSCCC Straightforward Fast Preparative Method for Isolation of Two Major Cytotoxic Withanolides from *Athenaea fasciculata* (Vell.) I.M.C. Rodrigues & Stehmann

**DOI:** 10.3390/plants13213039

**Published:** 2024-10-30

**Authors:** André Mesquita Marques, Lavínia de Carvalho Brito, Maria Raquel Figueiredo

**Affiliations:** Laboratório de Produtos Naturais (TecBio), Farmanguinhos, FIOCRUZ Foundation, Rua Sizenando Nabuco 100, Rio de Janeiro 21041-250, RJ, Brazilmraquelf6@yahoo.com.br (M.R.F.)

**Keywords:** *Aureliana*, cancer, CCC, aurelianolides, separations

## Abstract

*Athenaea fasciculata* belongs to the Solanaceae family and is a promising source of cytotoxic withanolides known as aurelianolides A and B. In the last years, the pharmacological studies of these aurelianolides on different leukemia cell lines have stimulated new studies on their potential as alternative candidates for new lead anticancer drugs. However, the obtention of these two pure compounds by traditional preparative is a costly and long time-consuming process, which is performed in several steps. This study aimed to propose a straightforward approach for isolating aurelianolides A and B using high-speed countercurrent chromatography (HSCCC). In this study, among 10 different solvent systems, the system composed of *n*-hexane/ethyl acetate/methanol/water 3:6:2:1 (v/v/v/v) was chosen for optimization. This HEMWat system was optimized to 4:8:2:4 (v/v/v/v) and chosen for HSCCC separation in a tail-to-head elution mode. After the HSCCC scale-up procedure, a withanolides mixture (200.0 mg) was separated within 160 min in a single-step purification process. In total, 78.9 mg of aurelianolide A (up to 95.0% purity) and 54.3 mg of aurelianolide B (up to 88.5% purity) was obtained by this fast sequential liquid–liquid partition process. The isolated withanolides were identified by ^1^H and ^13^C NMR spectroscopy (this method has proven to be faster and more efficient than classical procedures (CC and Prep-TLC)).

## 1. Introduction

Cancer is the second leading cause of mortality worldwide, with projections indicating a transition from 20 million new cases in 2020 to 32.6 million in 2045 [1]. Various treatment modalities for this disease encompass surgical interventions, radiotherapy, biological therapy, and chemotherapy. However, chemotherapy frequently induces cytotoxic effects due to its non-selective nature, resulting in adverse side effects and drug resistance [2]. Despite these inherent limitations, natural products have long been recognized in pharmaceutical research as a promising source for discovering novel therapeutic agents for the management of various cancer types [3].

In this regard, withanolides stand out as a promising class of anticancer compounds. These compounds are steroidal triterpene lactones characterized by an ergostane skeleton composed of 28 carbon atoms, which can present a δ-lactone ring formed by oxidation at C-26 and C-22 (type I) or a γ-lactone ring formed by oxidation at C-26 and C-23 (type II). Structure–activity relationship (SAR) studies have revealed that the cytotoxic activities of these compounds are associated with the presence of a β-unsaturated ketone (ring A), a 5β, 6β-epoxide ring (ring B), along with a nine-carbon side chain substituted at C-17, featuring an α, β-unsaturated-δ-lactone. The structural classification of natural withanolides and their biological activities, particularly their cytotoxic effects, have been extensively studied [4,5,6,7,8].

**Figure 1:**
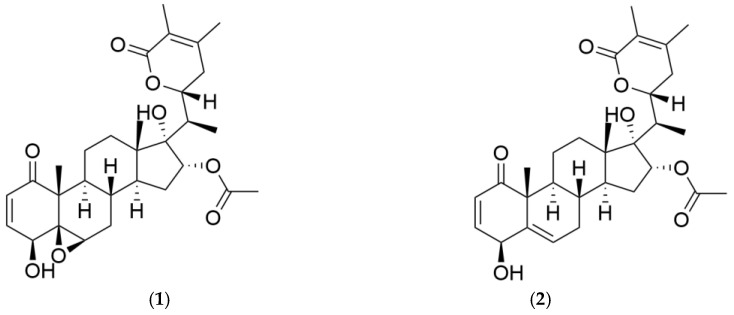
Structures of the withanolides aurelianolides A (**1**) and B (**2**).

In this study, we highlight *Athenaea fasciculata* (Vell.) I.M.C. Rodrigues & Stehmann, a species of the Solanaceae family [9], also known in the past as *Aureliana fasciculata* var. *fasciculata*. This Brazilian native species is characterized by the presence of withanolide compounds known as aurelianolides A and B (Figure 1).

Recently, the cytotoxicity of aurelianolides A and B isolated from *A. fasciculata* was assessed across a panel of leukemia cell lines. The results showed that aurelianolide A, the epoxide derivative, exhibited greater cytotoxic activity against MOLT-4 cells (IC_50_ = 1.17 μM), while aurelianolide B demonstrated a stronger effect in Jurkat cells (IC_50_ = 2.25 μM). Both compounds induce apoptosis with caspase activation in all tested cell lines without necrosis [10]. Besides their anticancer activity, both withanolide compounds have also demonstrated in silico activity against the intracellular forms of *Trypanosoma cruzi*, with results comparable to those of benzimidazole (Bz) and without the toxicity and mutagenicity associated with Bz [11]. In addition, both compounds also showed significant leishmanicidal effects. In silico assessments of absorption, distribution, metabolism, excretion, and toxicity (ADMET) parameters revealed similarities to miltefosine, with no observed hepatotoxicity [12].

The search for these bioactive compounds, which exhibit a plethora of pharmacological properties, involves their isolation on a preparative or semi-preparative scale from plant biodiversity and represents a primary goal of natural product chemistry [13]. Identifying suitable methodologies for the separation, isolation, and purification of these compounds has been a significant challenge in this area [14]. In recent decades, chromatographic methods have become pivotal tools for determining the phytochemical profiles of complex extracts [15]. Techniques such as column chromatography (CC) can facilitate the isolation of natural compounds, particularly on a large scale from plant extracts, even when dealing with compounds that have similar chemical and physical properties [16]. However, their utility is limited by several significant disadvantages, including high solvent consumption, time-intensive processes, low recovery due to irreversible adsorption, and poor reproducibility [14].

Many chromatographic methods based on irreversible adsorption have been used for isolating triterpenes. However, they often result in multiple purification stages and low final process yields [17]. High-speed countercurrent chromatography (HSCCC) is a separation technique that relies on liquid–liquid partitioning and is distinguished from conventional chromatographic methods by the absence of solid supports as a stationary phase. This feature prevents irreversible adsorption of compounds and sample depletion. Component isolation in a mixture is achieved by partitioning a solute between the two phases of a biphasic solvent system [18,19]. HSCCC is highly suitable for preparative separation, offering numerous benefits, such as minimal solvent consumption, high recovery rates, excellent reproducibility, and the capacity to handle substantial sample loads. Additional advantages include the versatility of solvent systems (both polar and nonpolar) and its advanced capabilities in partition efficiency and separation speed, making it a highly predictive and economical technique [14,20,21]. Furthermore, its low chemical and instrumental costs make HSCCC more cost-effective compared to other chromatographic methods. Due to its efficiency, HSCCC has been successfully employed for separating a wide variety of natural products, including numerous terpene compounds [22,23,24,25]. In our previous studies, the fractionation of the dichloromethane partition, obtained from the ethanol leaves extract, was conducted using several chromatographic processes to separate the two major active withanolides from various unknown substances [10,12,26]. The preliminary separation using silica gel column chromatography and Sephadex-LH20 resulted in enriched fractions that still contained impurities and a mixture of withanolides. Final purification of these compounds was achieved only after several preparative TLCs and a continuous recrystallization process, which required a lengthy, costly, and time-consuming effort.

In this study, we introduce a fast preparative method to separate the major withanolide compounds of *A. fasciculate*, from an enriched fraction using a single CCC run. Initially, the dichloromethane fraction (2.0 g) was cleaned using Sephadex-LH20 to remove chlorophyll and several impurities. The target compounds were then concentrated using a silica gel column with silica gel 60, eluted with a 30% *n*-hexane/ethyl acetate gradient solvent. Subsequently, the fast purification of aurelianolides A and B was accomplished in a single step using HSCCC in just 2.6 h.

Given the high interest in the pharmacological properties of aurelianolides A and B, this study aimed to propose a straightforward approach using the HSCCC technique for the preparative-scale isolation and purification of these two major bioactive compounds from *Athenaea fasciculata*.

## 2. Results and Discussion

### Separation Procedure and Scale-Up

The stability of the two-phase solvent system after reaching hydrostatic equilibrium significantly influences the HSCCC procedure, as sample loading is limited by the solvent volume in the loop injection and the stationary phase’s solubilization capacity. Excessive sample loading can easily lead to the loss of the stationary phase, which reduces the interaction between the analyte and the two immiscible phases, ultimately resulting in poor purity of the obtained target compound [27]. In this study, the effects of sample loading were evaluated in a scale-up experiment using 50.0 mg and 200.0 mg of WITAB sample to assess the purity of the target compounds. In both cases, the purity of the obtained target fractions was comparable, achieving up to 92% purity for both compounds, regardless of the sample loading. In the first separation process, 50.0 mg of WITAB was used as the initial point for optimizing sample loading. Due to the high retention of the stationary phase, the selected solvent remained stable throughout continuous separation and purification. Total sample solubilization was achieved by performing multiple injections of the upper and lower phases separately, as previously described in the section on the scale-up and separation procedure topic.

**Figure 2:**
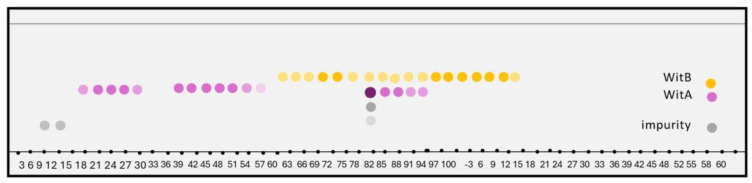
TLC figure scheme of HSCCC WITAB separation process 1. WitA: aurelianolide A. WitB: aurelianolide B. The intensity of the dots color pattern of the samples reflects the concentration of the sample at each application point. Dots with more intense colors correspond to more concentrated fractions obtained in the separation process.

The WITAB sample was introduced into the HSCCC using a solvent system composed of n-hexane–ethyl acetate–methanol–water in a ratio of 4:8:2:4 (v/v/v/v) in tail-to-head elution mode, with the organic phase acting as the stationary phase. The chemical profile of this HSCCC separation process, as determined by TLC, is shown in Figure 2. Based on the TLC profiles, similar fractions were combined, resulting in four main sample fractions. Fractions 21–30 and 39–54 yielded 19.5 mg with a purity of 97.2% and were combined as sample (**I**). Sample (**II**) consisted of fractions 60–79, yielding 7.8 mg with a purity of 95.8%. Sample (**III**) was characterized as a mixture of two substances, in the fractions 82–95 comprising 77.0% and 17.0%, yielding 9.6 mg. Sample (**IV**) consisted of 6.75 mg obtained from the last fractions (96–116), with a purity of 92.0%. The total mass recovery for this process was 82.5%.

**Figure 3:**
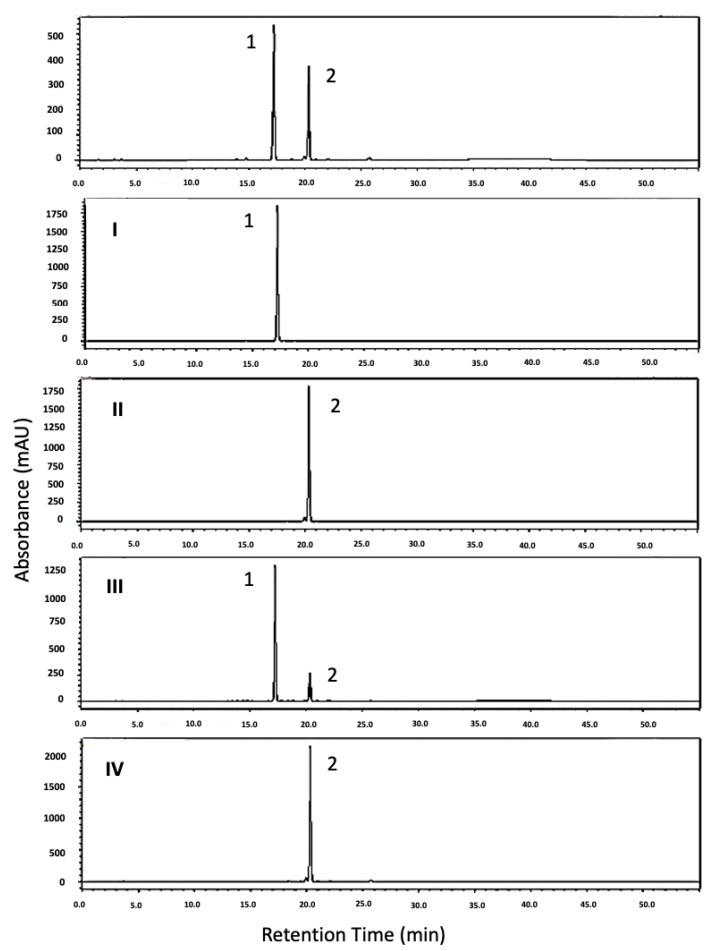
HPLC chromatogram of withanolide mixture (WITAB) and HSCCC main samples (I–IV) obtained from WITAB mixture separation. Compound 1-aurelianolide A and compound 2-aurelianolide B.

All the combined fractions obtained from HSCCC separation process 1 were analyzed by HPLC (Figure 3). Among them, sample (**I**) was identified as aurelianolide A, while samples (**II**) and (**IV**) corresponded to aurelianolide B. Sample (**III**) was found to be a mixture, with aurelianolide A as the major compound. The purity of the samples was determined by HPLC, revealing purities of 97.2% for sample (**I**), 95.8% for sample (**II**), 77.0% for sample (**III**), and 92.0% for sample (**IV**). Further identification was performed using ESI-MS and ^1^H and ^13^C 1D and 2D NMR analyses, which confirmed the identity of the target compounds previously reported [10,26], as detailed in the Supplementary Materials (Figures S1–S14; Tables S1 and S2).

**Figure 4:**
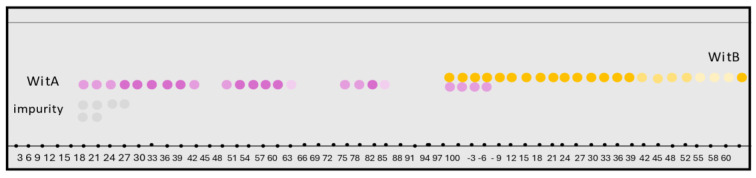
The TLC scheme of HSCCC WITAB scale-up separation process 2. WitA: aurelianolide A. WitB: aurelianolide B. The intensity of the dots color pattern of the samples reflects the concentration of the sample at each application point. Dots with more intense colors correspond to more concentrated fractions obtained in the separation process.

Under the optimized conditions, an increased amount of WITAB sample (200.0 mg) was separated using HSCCC separation process 2, following the same established procedures previously described. The chemical profile of this HSCCC separation process was performed by TLC (Figure S2). Based on the TLC profiles, similar fractions were combined, resulting in six main samples. Sample (**I**), derived from fractions 30–39, yielded 18.7 mg with a purity of 95.1%. Sample (**II**), from fractions 60–79, yielded 34.0 mg with a purity of 95.6%. Sample (**III**), from fractions 72–85, yielded 26.2 mg with a purity of 94.0%. The combined fractions 98–106 were characterized as a mixture of two substances, yielding 6.0 mg (sample **IV**). Sample (**V**), from fractions 109–115, yielded 19.1 mg with a purity of 85.0%, while sample (**VI**), from fractions 116–126, yielded 23.5 mg with a purity of 88.7%. Finally, sample (**VII**), obtained from the last fractions 127–155, yielded 11.7 mg with a purity of 92.0%. The total mass recovery for this process was 85.6%. The TLC analysis of all fractions obtained from the HSCCC WITAB scale-up separation process is shown in Figure 4.

**Figure 5:**
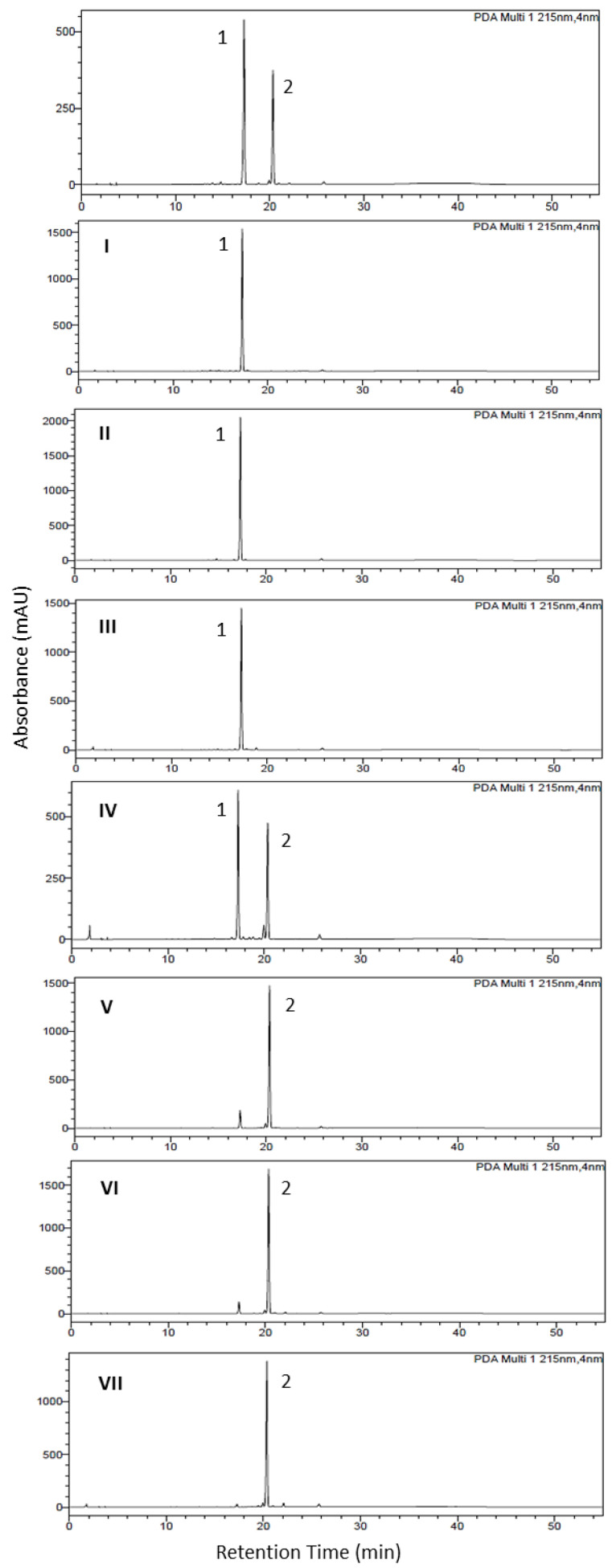
HPLC chromatogram of withanolides mixture (WITAB) and HSCCC scale-up main fraction’s separation (I–VII) from WITAB mixture. Compound 1-aurelianolide A and compound 2-aurelianolide B.

All the combined fractions obtained from the HSCCC scale-up separation process 2 were analyzed by HPLC, as shown in Figure 5. Among them, samples (**I**) from fractions 30–39, sample (**II**) from fractions 50–69, and sample (**III**) from fractions 72–85 were all identified as aurelianolide A. Samples 98–106 (sample **IV**) were characterized as a withanolides mixture quite similar to the original sample. In contrast, the remaining samples—sample (**V**) from fractions 109–115, sample (**VI**) from fractions 116–126, and sample (**VII**) from the last fractions 127–155 corresponded to aurelianolide B. The purity of the samples was also assessed by HPLC, indicating that the HSCCC scale-up separation process 2 was highly efficient for the purification of aurelianolide A, with samples (**I**), (**II)**, and (**III**) achieving purities of up to 94.0%. In comparison, the purity levels of samples (**V**), (**VI**), and (**VII**), which were associated with aurelianolide B, were 85.0%, 88.7%, and 92.0%, respectively. Further identification was conducted using ESI-MS and ^1^H and ^13^C 1D and 2D NMR analyses, corresponding to the target compounds previously reported [10,26] as described in the Supplementary Materials (Figures S1–S14; Tables S1 and S2).

To date, no studies have been published regarding the separation of withanolides by HSCCC. However, several works have reported the efficiency of the CCC technique for the preparative separation of triterpenes derivatives compared to traditional silica gel column chromatography and dextran gel column chromatography. Conventional separation methods utilizing liquid, open columns often involve more complicated experimental procedures, leading to high consumption of organic solvents, prolonged separation times, irreversible sample adsorption, and low solute recovery [28]. Pre-concentration and pre-separation of complex mixtures containing triterpenes across a wide range of polarities are commonly employed in various purification process designs. For instance, Zhu et al. [29] utilized AB-8 macroporous resin for the pre-purification and concentration of triterpenes, successfully separating corosolic and nigranoic acids—two acid triterpenes isolated from *S. chinensis*—using a CHCl₃–*n*-BuOH–MeOH–water solvent system. The separation of 100 mg of the enriched fraction was completed within 300 min using HSCCC. Another alternative method for the rapid preparative separation of acid triterpenes involves employing pH-zone-refining during the elution process. This approach demonstrates that the efficiency of the CCC technique is also applicable to the separation of structurally isomeric pentacyclic triterpene acids, such as oleanolic and ursolic acids. Both compounds were isolated at high purity levels from three different species using HSCCC with an *n*-Hex–CH₂Cl₂–MeOH–water solvent system, completing the separation of 100 mg of crude extract in 350 min [30].

Demonstrating the versatility of HSCCC and its wide polarity range, Ping et al. [31] showcased the technique’s potential for separating nonpolar triterpenes using an *n*-Hex-ACN 1:1 (v/v) solvent system from the fungus *Inonotus obliquus*. In this study, the triterpene extraction was performed by NPCE, yielding 17.41 mg/g of an enriched triterpene fraction. Four nonpolar triterpenoids were obtained in a single-step separation, achieving purities exceeding 95% within 350 min of HSCCC run time. In another study, Du et al. [32] performed the separation of several triterpene aglycones and glycosides across a wide range of polarities using an *n*-Hex/BuOH biphasic system. The authors employed a mobile phase gradient elution mode to separate 600 mg of a polar crude extract from *Centella asiatica* using HSCCC over 520 min.

Unlike the works cited, our developed method presented an efficient separation with less solvent consumption and reduced execution time, being easily reproduced. Unfortunately, separation methods of withanolides by CCC are rare in the literature to date. By comparing our separation method with others developed for the separation of triterpenes, most of the works involving the separation of triterpenes mentioned use pre-separation and concentration of triterpenoids, as was carried out in our work. The removal of minor interferents contributes during the cleanup process, reducing the number of metabolites that would coelute during separation and thus increasing the purity of the isolated samples. Our work contributed to solving and controlling a common problem related to triterpenes, which is the issue of solubility of these compounds. By separating and injecting the different phases (upper and lower) separately, and also with the addition of methanol in each phase, it was possible to increase the solubility and, thus, the sample injection capacity. This procedure contributes to obtaining significant results in quantitative terms in a short period of run time because our separation process demonstrated a good capacity to separate similar structures in less than 3 h of the separation process, well below the presented methods in the literature. In addition, it is also worth highlighting the reduced use of solvent systems used in our withanolides separation. In total, 1 L of HEMWat quaternary solvent system is consumed. Many authors emphasized the valuable attributes of preparative HSCCC techniques, particularly their high sample loading capacity and the absence of irreversible adsorption effects of analytes to the solid phase, in contrast to preparative HPLC. Given the versatility of solvents that can be utilized, such as chloroform, methanol, and water, CCC can also effectively separate various polar triterpene compounds, including saponins or glycosylated withanolides for example.

## 3. Materials and Methods

### 3.1. Reagents and Materials

All organic solvents used for HSCCC and HPLC were of HPLC grade and were supplied by Tedia Brazil (Rio de Janeiro, Brazil). Water was purified using the Milli-Q water system from Millipore (Bedford, MA, USA). The chromatographic process was performed using high-purity solvents and materials, including Silica gel 60 G from Merck (Darmstadt, Germany) and Sephadex LH-20 (Sigma-Aldrich, St. Louis, MO, USA).

### 3.2. Botanical Material

*Athenaea fasciculata* (Vell.) Sendtner was collected in Simão Pereira (MG) and identified by Dr. Rita de Cassia Almeida-Lafetá (UFRJ). The exsiccate was deposited in the Herbarium of UFRJ, Rio de Janeiro, Brazil, under number 40829. This species is registered in the Genetic Heritage (SisGen) database under the number AB5D582.

### 3.3. Plant Extraction

The fresh leaves of *Athenaea fasciculata* (1.2 kg) were dried in an oven with air circulation at 40 °C for 48 h. After drying, the material (465.0 g) was reduced to small fragments in a knife mill and extracted by dynamic maceration at room temperature with ethanol for 48 h. The extract was filtered and concentrated under reduced pressure in a rotary evaporator, yielding 40.0 g. The dry extract was suspended in MeOH/H_2_O 7:3 (v/v) to obtain the liquid–liquid partitions in sequence with *n*-hexane, dichloromethane, ethyl acetate, and butanol. In this work, only the partition in dichloromethane (AFFD) 2.6 g, rich in aurelianolide A (WitA) and aurelianolide B (WitB), was processed.

### 3.4. HPLC Analysis and Identification of HSCCC Peak Fractions

The WITAB mixture and the HSCCC sample analyses were conducted using HPLC with a Shimadzu system (Kyoto, Japan), which included an LC-10AD pump, SIL-10A as an automatic injector, and UV/Vis SPD-20AV as a detector, all controlled by CLASS LC-10, VP software, version 4.2. HPLC analysis was performed with a Synergi Polar RP-18 column (150 × 4.6 mm, 4 µm) from Phenomenex (Torrance, CA, USA), coupled with a refillable pre-column, filled with C-18 silica (150 µm particle size) from Grace (Deerfield, MA, USA). The mobile phase comprised acetonitrile and water in a 37:63 (v/v) ratio, with 1% orthophosphoric acid, operated in isocratic mode. The flow rate was set to 1.2 mL/min at 33 ± 1 °C, with a 20 µL injection volume and detection wavelength of 367 nm.

### 3.5. AFFD Chlorophyll Cleanup

The fraction AFFD (2.0 g) was chromatographed on a Sephadex LH-20 column using methanol/chloroform in a 3:1 ratio as a mobile phase to rapidly remove chlorophyll and some impurities. Thin-layer chromatography (TLC) on silica gel 60 F_254_ was employed to monitor the collected fractions, using an eluent solution of 2% methanol in dichloromethane and 2% CH_2_Cl_2_ in H_2_SO_4_ as the revealing agent, applied on a hot plate at 110 °C. Only the later fractions containing aurelianolides A and B, as determined by TLC similarity, were selected and combined, resulting in a yield of 1.2 g of a withanolide-enriched sample.

### 3.6. Aurelianolide A and B’s Pre-Concentration

**Figure 6:**
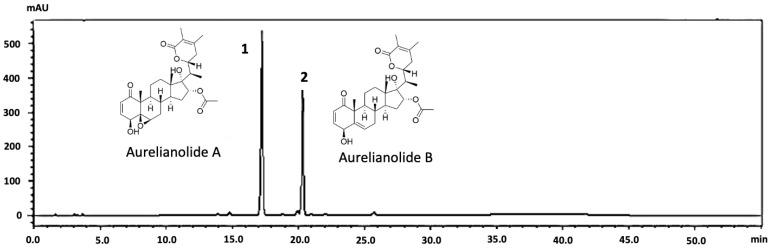
HPLC chromatogram of aurelianolides A and B mixture (WITAB).

The sample obtained from the chlorophyll cleanup process, containing 1.2 g target compounds, was separated by chromatography column on silica gel (SiO_2_ 60—0.063–0.200 mm) by elution with increasing polar solvents gradient as follows (*n*-hexane, ethyl acetate, ethyl, and MeOH). In total, 52 fractions were obtained and evaluated by TLC under the same conditions described before to result in a single sample composed of a mixture of the aurelianolides A and B (WITAB) (700.0 mg) (Figure 6).

### 3.7. High-Speed Countercurrent Chromatography (HSCCC)

#### 3.7.1. Selection of Two-Phase Solvent System

The choice of solvent systems was based on the distribution behavior of the target compounds between the two immiscible phases of tested solvent systems. The distribution coefficient is calculated by the formula: *K_D_* = SP/MP, where *K_D_* is the distribution coefficient, SP and MP represent the HPLC peak areas of the aurelianolides A and B in the stationary and mobile phases, respectively. As a starting point, 10 different two-phase solvent systems chosen from the literature sources were evaluated to analyze the behavior of the compounds in the two immiscible phases (Table 1). The solvent system composed of *n*-hexane/ethyl acetate/methanol/water in a proportion of 3:6:2:1 was chosen for optimization based on the best target compound distribution. The HEMWat two-phase solvent system was modified to cover a wide range of polarities by changing their component volume in different proportions, and the solvent system composed of *n*-hexane/ethyl acetate/methanol/water in a proportion of 4:8:2:4 was chosen based on the best target compounds distribution (Table 2). A small amount of the WITAB (5.0 mg) was dissolved in a test tube containing each solvent system. The tubes were vigorously shaken and waited until the two phases were allowed to settle. The distribution of the compounds into the upper and lower phases was first estimated by thin-layer chromatography (TLC, silica gel 60 F_254_ nm) by using equal volume samples and using dichloromethane/methanol (2%) as the eluting solvent system. The final elution process on TLC was observed under a UV lamp at 254 nm and 365 nm. To assist the visual estimation of the relative distribution of the compounds in each phase, a ceric sulfate/ethanol reagent (0.2%) was sprayed over the sample, followed by heating under 110 °C on a hot plate.

#### 3.7.2. HSCCC Equipment and Separation Procedure

To perform HSCCC separation, the solvent system with the best distribution coefficient was chosen. After testing the two-phase solvent systems optimization, the HEMWat mixture in the proportion of 4:8:2:4 (v/v/v/v) showed the best efficiency for HSCCC separation of the target compounds and was chosen for the separation process. The equipment used was a countercurrent chromatograph, model Quattro HT-Prep model (AECS, Bridgend, S. Wales, UK), consisting of four stainless steel 122 mL coils, an HPLC pump (model V10STF01Rev16 series II, Lab Alliance, State College, PA, USA), a low-pressure injection valve (Rheodyne 5020, Cotate, CA, USA), and a PTFE 5 mL sample loop was used. The rotation speed used was 860 rpm. This system was coupled to a fraction collector (model FC203B, Gilson Inc., Middleton, WI, USA) programmed to collect at 1.0 min intervals. Appropriate volumes of the solvent system *n*-hexane/ethyl acetate/methanol/water 4:8:2:4 (v/v/v/v) were vigorously hand-mixed in a separator funnel and separated after settling time. The upper and lower phases were separately transferred to flasks and degassed in an ultrasonic bath for 20 min. The coil was entirely filled with the stationary phase of the solvent system with no rotation applied. Isocratic elution was conducted in a head-to-tail manner (the upper phase was used as the stationary phase). The coil rotation was then settled at 860 rpm, and the lower phase was pumped at a flow rate of 2.5 mL/min. The system was allowed to attain hydrodynamic equilibrium prior to the sample injection. In total, 75.0% of the stationary phase was retained.

### 3.8. Scale-Up and Separation Procedure

#### Optimization of Separation and Purification Conditions

In CCC separations, the success of the process relies heavily on the appropriate selection of a two-phase solvent system that provides an ideal range of distribution coefficients (K*_D_*) for the target substances. According to the literature, the optimal K*_D_* values for separating the target compounds should range between 0.5 and 2.0. Small K*_D_* values lead to fast elution and a loss of resolution of the separation process, while large K*_D_* values require excessive amounts of solvent and extended run times, resulting in reduced efficiency [21]. In this study, 10 different two-phase solvent systems were tested. Based on the best K_D_values for both target compounds, the system composed of *n*-hexane–ethyl acetate–methanol–water in a 3:6:2:1 (v/v/v/v) ratio, using head-to-tail elution mode, was optimized by HSCCC (Table 1).

Due to its versatility and wide range of polarity, the *n*-hexane–ethyl acetate–methanol–water solvent system (HEMWat) is broadly recognized for its effectiveness in CCC separations and is frequently employed for the isolation of a wide range of natural compounds [23]. The optimization of this solvent system began with modifications to the HEMWat component ratios, followed by the measurement of K*_D_* values for the target compounds across these variations in this system. Thus, this two-phase system was adjusted to cover a broad range of polarities by altering the proportions of its components (Table 2). Among the systems tested, despite having K*_D_* values close to 1.0, systems 2, 3, 5, 7, and 9 were considered unsuitable for separating the target compounds. In these systems, the compounds exhibited nearly equal distribution between the two phases, with close K*_D_* values (K*_D_*_2_ − K*_D_*_1_ ≤ 0.2). This similar solubility and affinity of both compounds for the upper and lower phases typically result in poor resolution and separation. Conversely, the HEMWat solvent systems with ratios of 4:8:2:4 (v/v/v/v) (#6), 4:8:0.5:5 (#8), and 10:8:1:2 (#10) yielded suitable results based on the distribution of the target compounds between the phases.

Based on the K*_D_* values of aurelianolides A and B, the selected systems should theoretically be capable of separating the mixture. However, some of these systems may not provide an acceptable or efficient run time. In particular, systems 1 and 8 exhibited very high partition coefficient values (K*_D_*_1_ > 5), indicating that a significant portion of the compound would be retained in the stationary phase, leading to extended run times and a potential loss of resolution.

Beyond the K*_D_* value, successful separation also relies on the retention of the stationary phase. Higher retention of the stationary phase directly contributes to improved peak resolution [25]. Three systems demonstrated promising results, with stationary phase retention exceeding 70.0%, making them suitable for the separation of WITAB. The solvent system #10, with a volume ratio of 10:8:1:2 (v/v/v/v) in head-to-tail elution mode, exhibited 85.8% stationary phase retention. Meanwhile, solvent system #6, in a ratio of 4:8:2:4 (v/v/v/v) in tail-to-head elution mode, achieved 76.0% retention, and solvent #4, composed of 4:8:1:2 (v/v/v/v) in tail-to-head elution, retained 72.0% of the stationary phase, after reaching hydrodynamic equilibrium. Despite the suitable K*_D_* values observed for systems #4, #6, and #10, the volume ratios for systems #4 and #10 were considered unsatisfactory, as the mobile phase comprised a small volume (approximately 1/6 V/V) after settling time, indicating significant waste of the stationary phase. Therefore, solvent system #6, consisting of *n*-hexane–ethyl acetate–methanol–water in a volume ratio of 4:8:2:4 (v/v/v/v) in tail-to-head elution mode, was considered the most suitable and economical option for the preparative separation and purification of the target compounds and was selected for the subsequent separation process.

According to the results, the increasing of methanol and water volumes contributes to the equal distribution of the target compounds, as observed in systems #5 and #9. However, when methanol is decreased and water is increased, the polarity of the two phases is enhanced, leading to a tendency for compound 2 to be retained in the organic phase (Table 2). A high concentration of *n*-hexane increases the K*_D_* values for both compounds, resulting in greater retention in the stationary phase, as demonstrated in system #1.

It appears that modifications in the methanol/water ratios are crucial for achieving selectivity in the distribution of the target compounds. In system #5 (*n*-Hex–EtOAc–MeOH–H_2_O), 4:8:4:3 (v/v/v/v), the K*_D_*_1_ and K*_D_*_2_ values were found to be 1.3 and 1.2, respectively. Reducing the amount of methanol while increasing the water in the HEMWat system significantly alters the K*_D_* values of the target compounds. For example, while K*_D_*_1_ was 1.5, indicating nearly equal distribution between both phases, K*_D_*_2_ doubled to 2.6, suggesting that compound 2 is more hydrophobic and exhibits a preference for the organic phase. Consequently, the upper organic phase was selected as the stationary phase. The total fractionation time was 160 min, demonstrating that the HSCCC separation could be performed fast.

As a starting point, the method was developed using 50.0 mg of the withanolides mixture (WITAB) in the HSCCC separation process. However, unlike the initial tube tests, the increased sample amount was not completely dissolved in the biphasic solvent system. To resolve this problem, the two phases were separated, and 1.0 mL of methanol was added in both phases. After a few minutes of ultrasound, both upper and lower phases become completely soluble. To conduct the separation procedure, two injections were performed. First, the lower phase was injected in time “0”, followed by the upper phase injection after 60 min directly on flow. In total, 160 fractions were collected in 160 min. The first 120 fractions were collected with rotation “on” and the last 40 fractions with rotation “off” in extrusion mode. All the fractions were monitored by TLC. The similar fractions were reunited and analyzed by HPLC. The scale-up process was performed by injecting a WITAB sample (200.0 mg), which was dissolved, as mentioned before. In this process, a quadruple injection of the samples was performed on flow: two upper phase injections on time “0” and time “20”, followed by double lower phase injection on time “45” and time “65” directly on flow. A total of fractions (n = 160) of 2.5 mL each were collected in the span of 160 min. The first 120 fractions were collected with rotation “on” and the last 40 fractions with rotation “off”. All the fractions were monitored by TLC. The target fractions considered visually pure were reunited by similarity and analyzed by HPLC.

### 3.9. NMR Analyses

Nuclear magnetic resonance spectra of hydrogen and carbon (^13^C and ^1^H NMR) were obtained on a Varian device VNMRS-Gemini 400 spectrometer operating at a frequency of 400 MHz (^1^H)/100 MHz (^13^C) using CD_3_OD as solvent. Special techniques and bi-dimensional such as COSY, HMBC, and HSQC were also performed. The chemical shift values (δ) in dimensionless units were referred to as an internal standard (TMS), being represented in parts per million (ppm) of the applied frequency for each experiment and coupling constants (*J*) were measured in Hz. The obtained data were compared to the literature data [26].

## 4. Conclusions

This work demonstrates the rapid and preparative separation of the major cytotoxic withanolides, aurelianolides A and B, from *A. fasciculata*. HSCCC has proven to be a valuable tool for the purification of structurally similar triterpene lactones, achieving high purity levels in a single step within 160 min. The results indicate that the present method is feasible, economical, and efficient for the rapid separation and purification of bioactive withanolides or standards derived from natural products. One of the major advancements achieved in this study is the isolation of metabolites in substantial quantities in preparative mode, in contrast to conventional support-based chromatography, which often encounters issues related to irreversible adsorption to solid supports. This positions HSCCC as a less costly and time-consuming alternative for preparative separation.

## Figures and Tables

**Table 1:** Two-phase solvent system tested for HSCCC separation.

	Two-Phase Solvents	Volume Ratio	Partition Coefficient (*K_D_*)	
			*K_D_*_1_	*K_D_*_2_
1	*n*-Hex/AcoEt/MeOH/H_2_O	3:6:2:1	1.5	1.3
2	*n*-Hex/MeOH	2:1	12.0	15.0
3	CHCl_3_/MeOH/H_2_O	7:13:8	4.4	5.8
4	CHCl_3_/BuOH/MeOH	7:3:6:4	13.0	17.0
5	CHCl_3_/MeOH/H_2_O	2:2:1	6.6	7.5
6	AcoEt/BuOH/H_2_O	1:1:2	14.0	17.0
7	*n*-Hex/BuOH/H_2_O	1:10:5	6.5	14.5
8	*n*-Hex/BuOH/MeOH/H_2_O	1:1:1:1	12.0	4.0
9	*n*-Hex/EtOH/H_2_O	6:5:1	15.0	16.0
10	*n*-Hex/AcoEt/ACN	1:1:1	18.0	18.0

**Table 2:** Optimization of two-phase solvent system composed of *n*-hexane/ethyl acetate/methanol/water tested for withanolides HSCCC separation.

	HEMWat Two-Phase Solvent System			
	Volume Ratio	V^UP^/V_LP_	Partition Coefficient (*K_D_*)	
			*K*_1_	*K*_2_
I	3:6:2:1	1/1	1.1	1.3
1	10:5:2.5:1	6/1	5.0	7.0
2	3:8:4:2	2/3	1.4	1.6
3	4:10:5:2	1/3	1.1	1.2
4	4:8:1:2	6/1	3.7	1.9
5	4:8:4:3	1/1	1.3	1.2
6	4:8:2:4	3/2	1.5	2.6
7	4:8:1:5	3/2	1.7	1.9
8	4:8:0.5:5	3/2	5.0	2.3
9	4:8:5:5	1/1	1.3	1.1
10	10:8:1:2	6/1	1.3	2.4

V^UP^/V_LP_: volume of upper phase/volume of lower phase distribution.

## Supplementary Materials

The following supporting information can be downloaded at:https://www.mdpi.com/article/10.3390/plants13213039/s1, Figure S1: HRESIMS of aurelianolide A; Figure S2: ^1^H NMR spectrum of aurelianolide A; Figure S3: ^13^C RMN spectrum of aurelianolide A; Figure S4: DEPT 135 NMR spectrum of aurelianolide A; Figure S5: COSY NMR spectrum of aurelianolide A; Figure S6: HSQC NMR spectrum of aurelianolide A; Figure S7: HMBC NMR spectrum of aurelianolide A; Figure S8: HRESIMS of aurelianolide B; Figure S9: ^1^H RMN spectrum of aurelianolide B; Figure S10: ^13^C RMN spectrum of aurelianolide B; Figure S11: DEPT 135 NMR spectrum of aurelianolide B; Figure S12: COSY NMR spectrum of aurelianolide B; Figure S13: HSQC NMR spectrum of aurelianolide B; Figure S14: HMBC spectrum of aurelianolide B; Table S1: NMR spectra data of aurelianolide A (CD_3_OD) isolated from *A. fasciculata*; Table S2: NMR spectra data of aurelianolide B (CD_3_OD), isolated from *A. fasciculata*.
