# Supplementary material for: HSCCC Straightforward Fast Preparative Method for Isolation of Two Major Cytotoxic Withanolides from Athenaea fasciculata (Vell.) I.M.C. Rodrigues & Stehmann
Source: Plants (Basel). 2024 Oct 30;13(21):3039. doi: 10.3390/plants13213039 (PMC11548422; doi:10.3390/plants13213039)
Supplement: Supplementary file 1 [file plants-13-03039-s001.zip › plants-3235312-supplementary.pdf]

Supplementary material for:

A straightforward fast preparative method for isolation of two major cytotoxic withanolides from *Athenaea fasciculata (Vell.) I.M.C. Rodrigues & Stehmann:* A High-Speed Countercurrent Chromatography Approach

André Mesquita Marques[a*], Lavínia de Carvalho Brito[a], Maria Raquel Figueiredo[a]

[a]*Laboratório de Produtos Naturais (TecBio), Farmanguinhos, FIOCRUZ, Rua Sizenando Nabuco 100, Rio de Janeiro, RJ, 21041-250, Brazil*
E-mail address: andrefarmaciarj@yahoo.com.br (A.M. Marques).

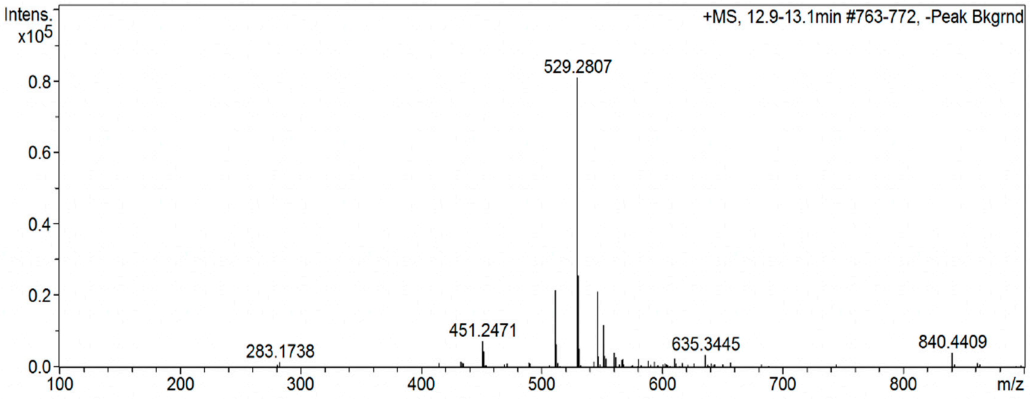

Figure S1: HRESIMS of aurelianolide A

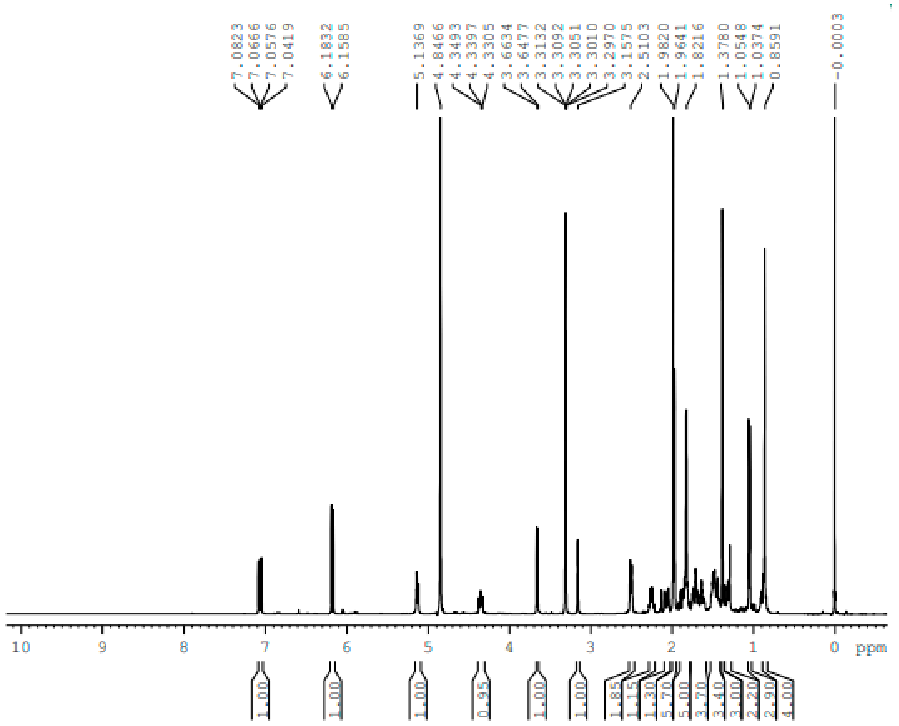

Figure S2: ^{1}H NMR spectrum of aurelianolide A

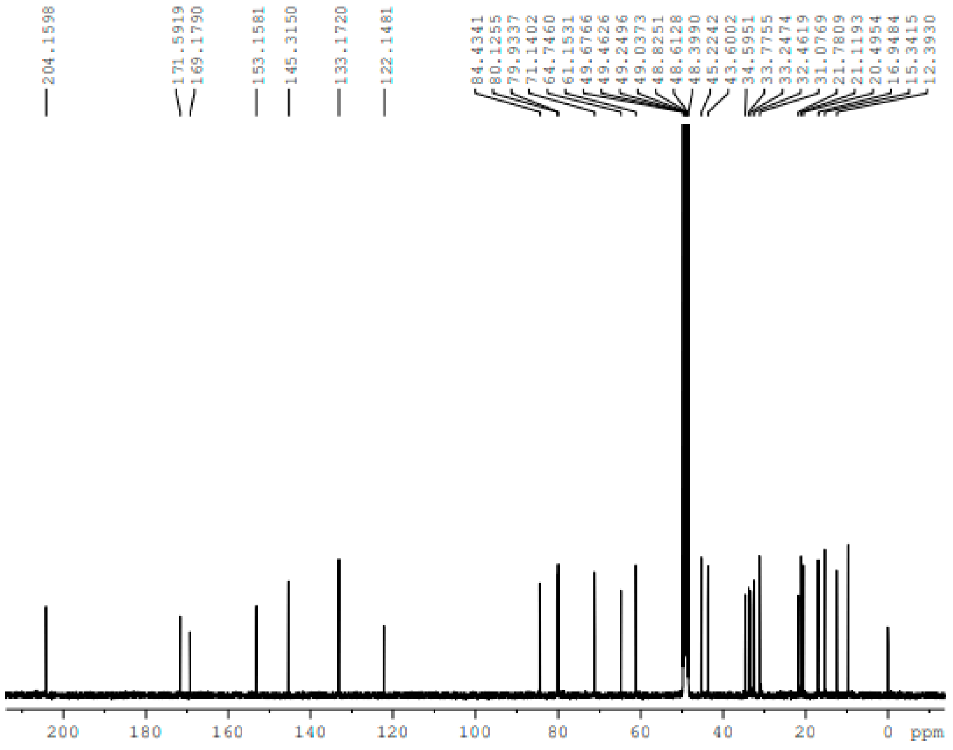

Figure S3: ^{13}C RMN spectrum of aurelianolide A

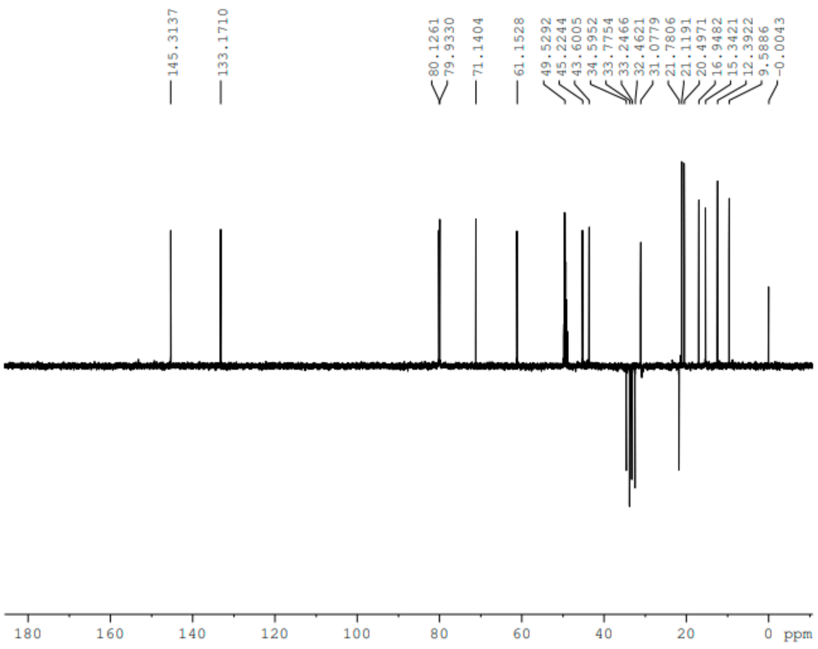

Figure S4: DEPT 135 NMR spectrum of aurelianolide A

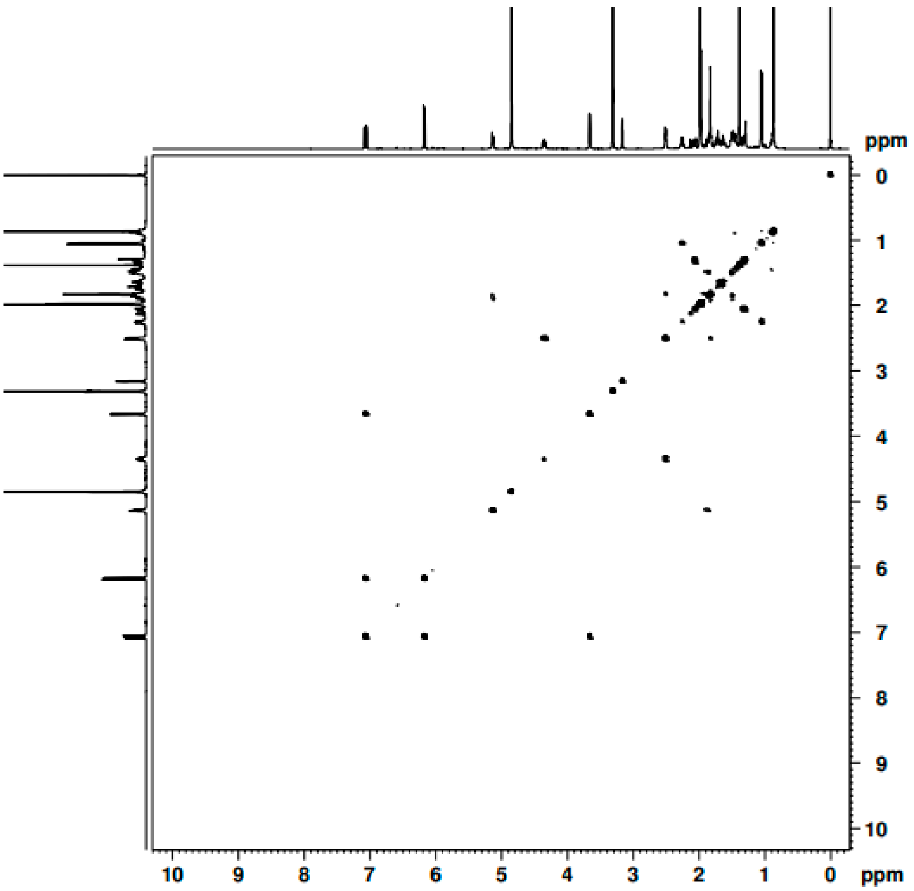

Figure S5: COSY NMR pectrum of aurelianolide A

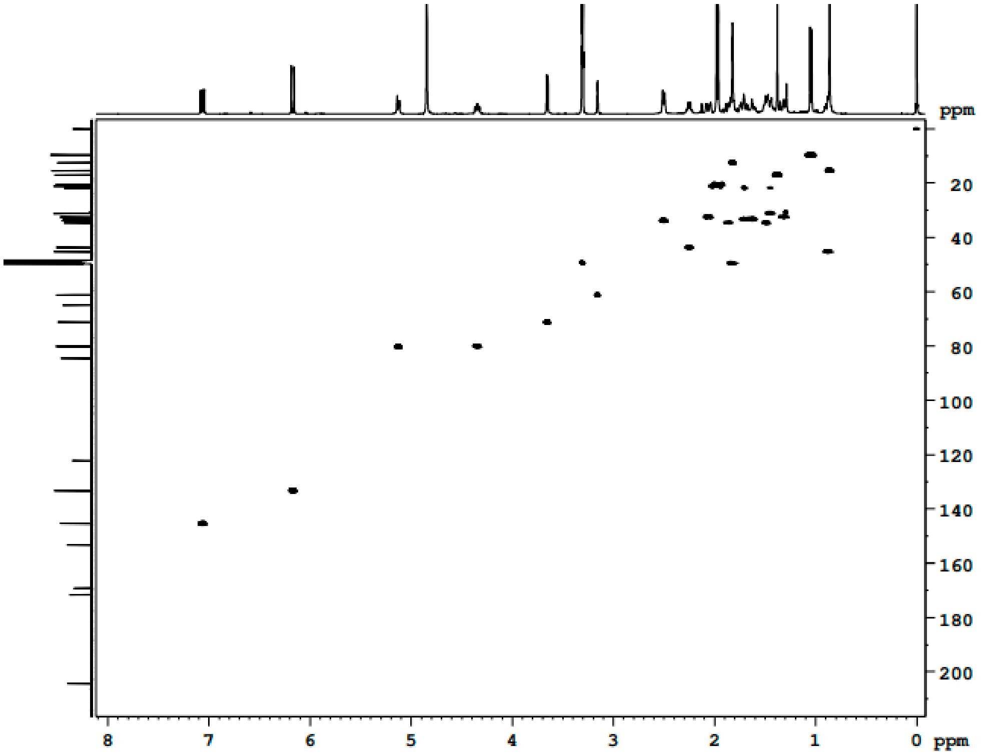

Figure S6: HSQC NMR spectrum of aurelianolide A

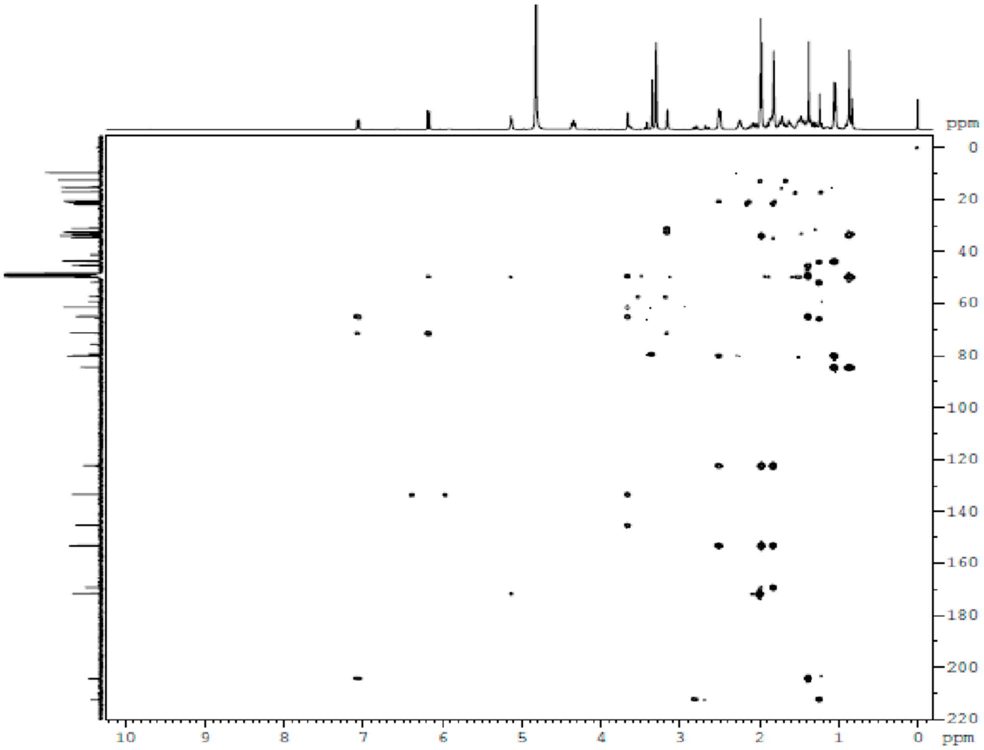

Figure S7: HMBC NMR spectrum of aurelianolide A

Table S1: NMR spectra data of Aurelianolide A (CD_3OD)

Posição	δ ^{13}C	δ ^{1}H	COSY	HSQC	HMBC
1	204.18	-	-	-	-
2	133.16	6.17	H3	C2	C4,C10
3	145.34	7.06	H2,H4	C3	C1,C5
4	71.14	3.66	H3	C4	C5, C6,C10
5	64.77	-	-	-	-
6	61.20	3.16	H8, H7a	C6	C4,C8
7	32.47	2.07 (H7a)	H6, H7b	C7	C11
		1.35 (H7b)	H7a	C7	-
8	31.09	1.50	H7b, H14	C8	C19
9	43.79	1.21	H7b	C9	C5, C9,C10
10	48.91	-	-	-	-
11	21.81	1.92 (H11a)	H11b	C11	-
		1.47(H11b)	H9,H11a,H12a e H12b	C11	-
12	33.26	1.71 (H12a)	H12b	C12	C18
		1.68 (H12b)	H12a	C12	-
13	49.05	-	-	-	-
14	49.59	1.75	H8,H15a,H15b	C14	-
15	34.60	1.89 (H15a)	H14,H15b	C15	-
		1.48 (H15b)	H15a	C15	C13,C14
16	79.93	5.12	H15a,H15b	C16	C do Ac-O
17	84.45	-	-	-	-
18	15.36	0.87	-	C18	C14, C15, C17
19	16.97	1.37	-	C19	C1, C5, C9,C10
20	43.62	2.25	H21,H22	C20	-
21	9.62	1.04	H20	C21	C17, C20,C22
22	79.26	4.35	H20	C22	-
23	41.23	2.81 (H23a)	H23b	C23	-
		2.65 (H23b)	H23a	C23	-
24	153.14	-	-	-	-
25	122.16	-	-	-	-
26	169.16	-	-	-	-
27	12.40	1.82	-	C27	C24, C25,C26
28	20.51	1.96	-	C28	C24,C25

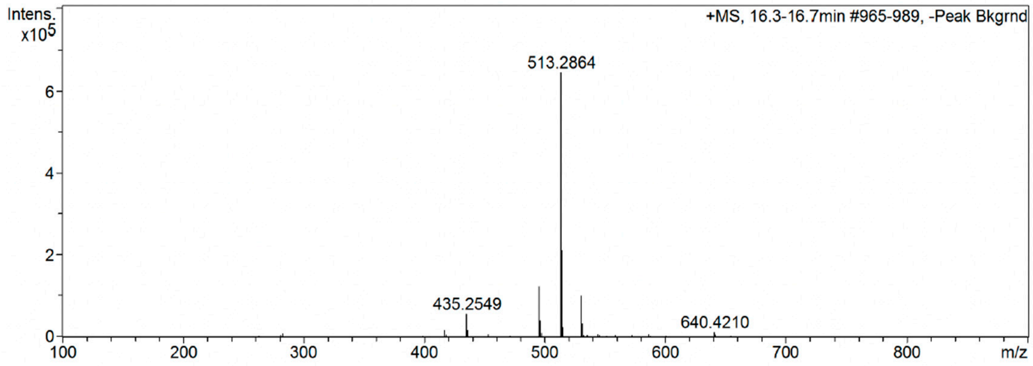

Figure S8: HRESIMS of aurelianolide B

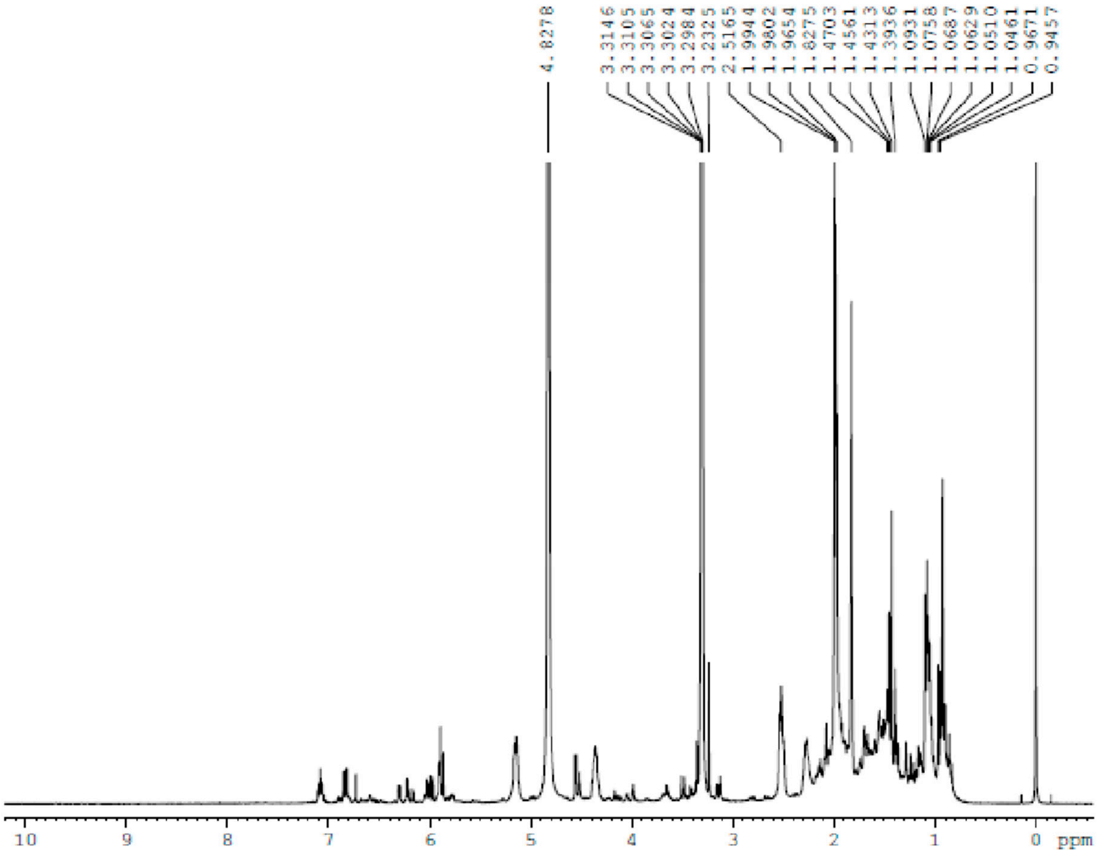

Figure S9: ^{1}H RMN spectrum of aurelianolide B

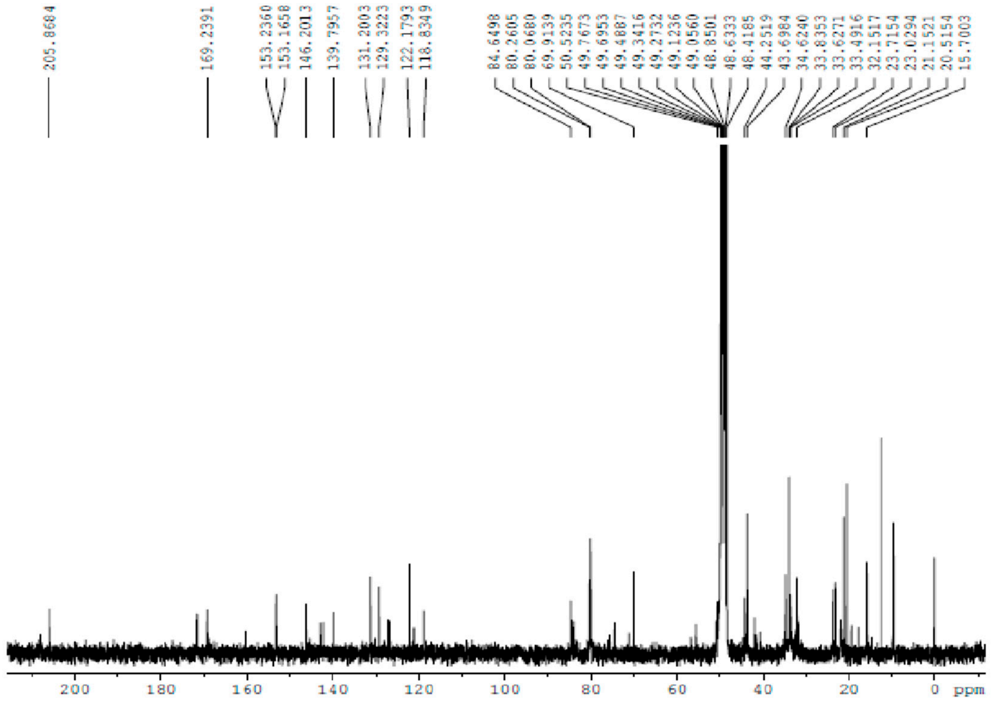

Figure S10: ^{13}C RMN spectrum of aurelianolide B

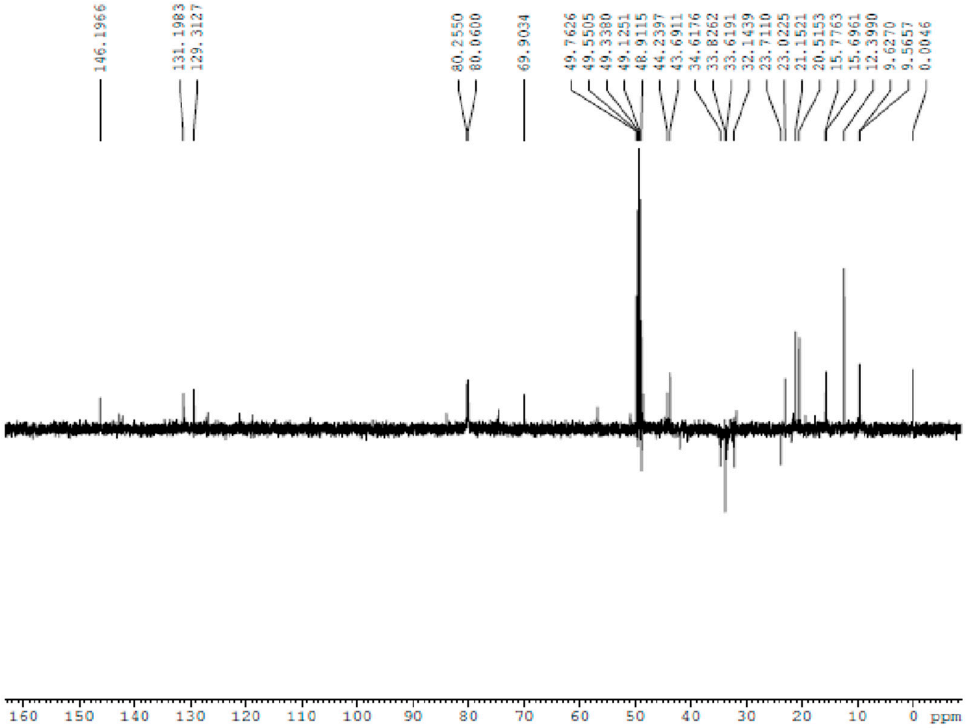

Figure S11: DEPT 135 NMR spectrum of aurelianolide B

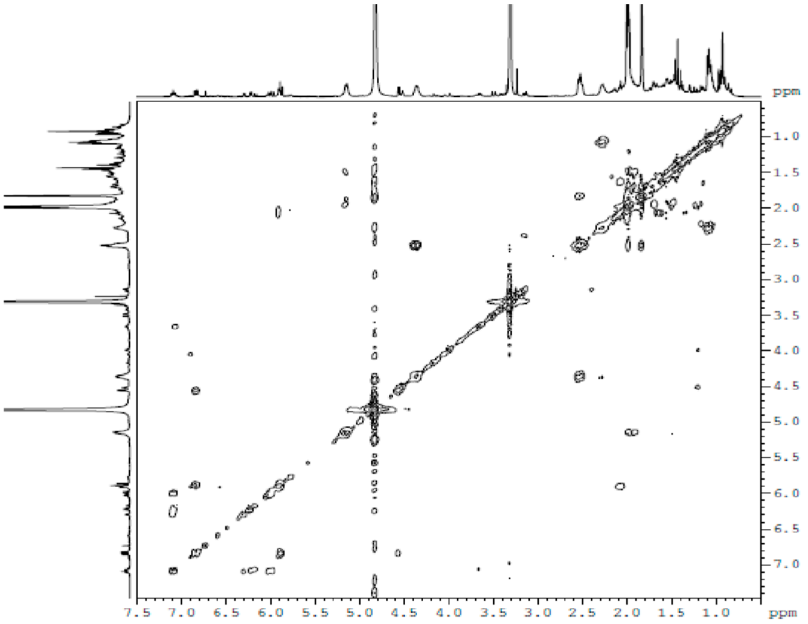

Figure S12: COSY NMR spectrum of aurelianolide B

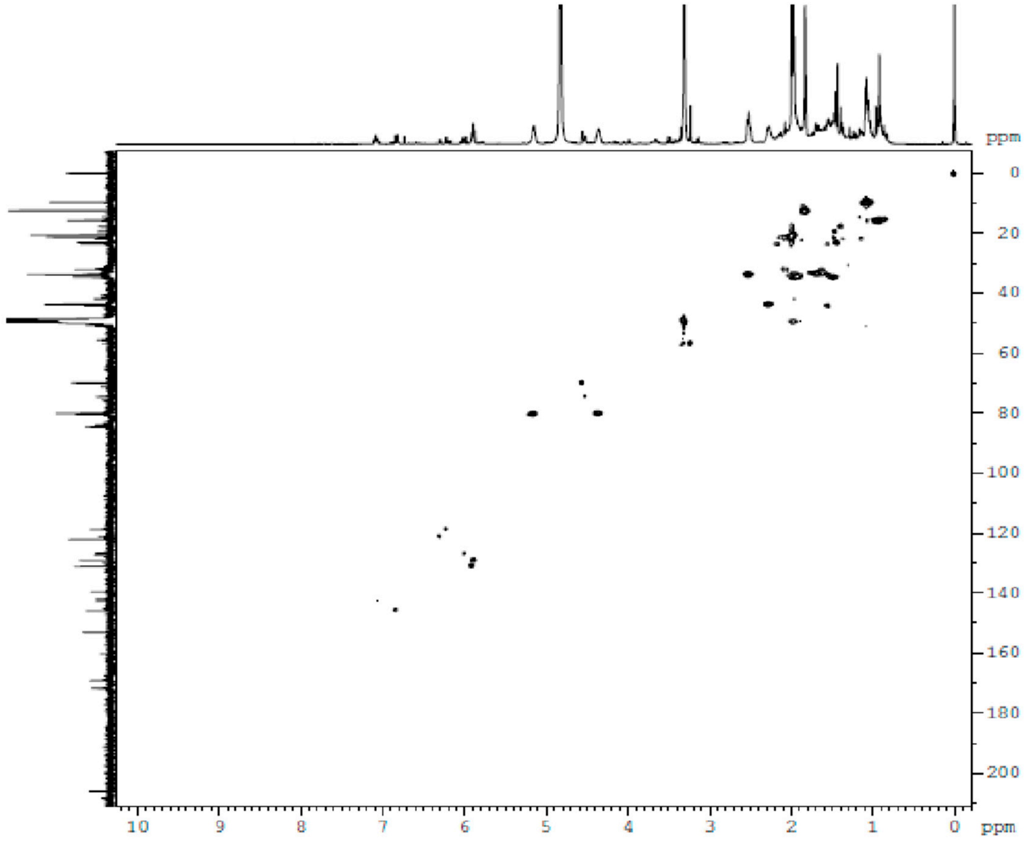

Figure S13: HSQC NMR spectrum of aurelianolide B

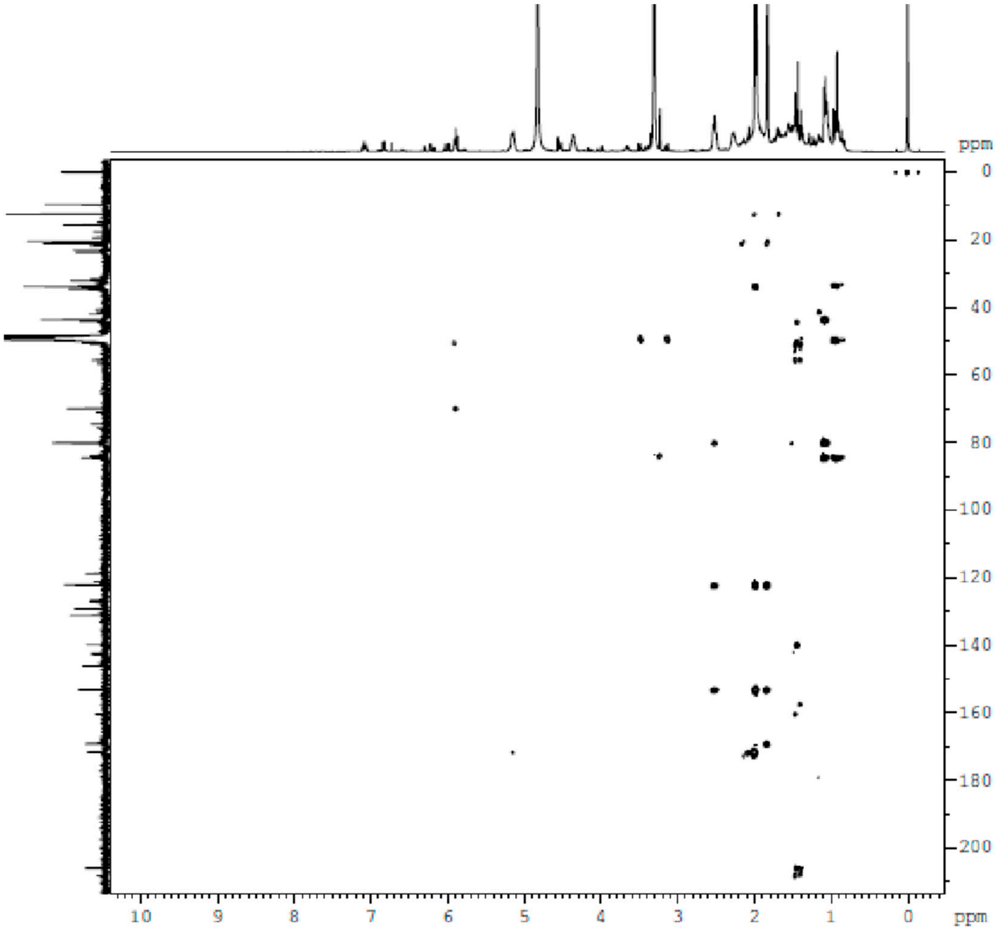

Figure S14: HMBC spectrum of aurelianolide B

Table S2: NMR spectra data of Aurelianolide B (CD_3OD)

Posição	δ ^{13}C	δ ^{1}H	COSY	HSQC	HMBC
1	205.87	-	-	-	-
2	129.32	5.88	H3		C4-C10
3	146.20	6.83	H2,H4	C3	-
4	69.91	4.55	H3	C4	-
5	139.80	-	-	-	-
6	131.20	5.89	H7a		C10
7	34.62	2.07 (H7a)			-
		1.46 (H7b)	H6,H8	C7	C1,C5
8	33.63	1.66	H15a	C8	C18
9	44.25	1.16	H8,H11a,H11b	C9	C9
10	50.52	-	-	-	-
11	23.71	2.15 (H11a)	-		C19
		1.55 (H11b)	H12a	C11	-
12	33.83	1.93 (H12a)	H11a,H12b		-
		1.69 (H12b)	H12a	C12	-
13	49.49	-	-	-	-
14	49.77	1.86	H15b	C14	-
15	34.62	1.90 (H15a)	H15b,H16		-
		1.50 (H15b)	H14,H15a	C15	-
16	79.98	5.15	H15a,H15b	C16	Ac-O
17	84.65	-	-	-	-
18	15.70	0.92	-	C18	C12, C14,C17
19	23.03	1.43	-	C19	C1, C5, C9,C10
20	44.24	2.27	H21,H22	C20	-
21	9.63	1.08	H20	C21	C17, C20,C22
22	80.27	4.36	H20,H23a	C22	-
23	33.84	2.51(H23a,H23b)	H22		C22, C24,C25
24	153.24	-	-	-	-
25	122.18	-	-	-	-
26	169.24	-	-	-	-
27	12.40	1.83	-	C27	C24, C25, C26,C28
28	20.51	1.98	-	C28	C23, C24,C25
Ac-O	171.72	-	-	-	-
Ac-CH_3	21.15	1.99	-	Ac-CH_3	Ac-O
